# Dietary cholesterol in alcohol-associated liver disease

**DOI:** 10.1097/IN9.0000000000000026

**Published:** 2023-05-03

**Authors:** Lin Jia

**Affiliations:** 1 Department of Biological Sciences, The University of Texas at Dallas, Richardson, TX, USA

**Keywords:** alcohol, alcohol-associated liver disease, cholesterol, cholesterol-lowering pharmacotherapies

## Abstract

There is an increasing prevalence of alcohol-associated liver disease (ALD) worldwide. In addition to excessive alcohol consumption, other nutritional factors have been shown to affect the initiation and progression of ALD. The emerging role of cholesterol in exacerbating ALD has been reported recently and the underlying mechanisms are discussed. In addition, the interplay between dietary cholesterol and alcohol on cholesterol metabolism is reviewed. Furthermore, we highlight the therapeutic potential of cholesterol-lowering drugs in managing the onset and severity of ALD. Finally, we suggest the future mechanistic investigation of the effect of cholesterol on insulin resistance and intestinal inflammation in the exacerbation of alcohol-induced cellular and systemic dysfunction.

## 1. Introduction

Alcohol-associated liver disease (ALD) is one of the leading causes of chronic liver disease worldwide. It has been projected that the annual age-standardized mortality due to ALD would increase by 75% by 2040, with the estimation that more than one million individuals in the United States could die from ALD between 2019 and 2040 ^[1]^. ALD involves a spectrum of hepatic pathological abnormalities ranging from simple steatosis to alcoholic steatohepatitis, which can progress to alcoholic cirrhosis and hepatocellular carcinoma. In addition to excessive alcohol consumption, several other risk factors have been identified to greatly affect the development and progression of ALD, such as gender, genetic background, smoking, preexisting liver disease, obesity, and nutrients ^[2,3]^. Recently, a population-based study showed that alcohol-drinking patients tend to intake more dietary cholesterol ^[4]^. It has been well-documented that cholesterol plays an important role in the development of severe liver damage ^[5]^ and cardiovascular disease ^[6,7]^. Thus, the investigation of the combined intake of alcohol and dietary cholesterol in disease development and progression is warranted.

## 2. The effect of dietary Cholesterol on alcohol-metabolizing enzymes

More than 90% of ingested alcohol is metabolized in the liver. Alcohol dehydrogenase (ADH) and cytochrome P450 2E1 (CYP2E1) are two major enzymes involved in the oxidative metabolism of alcohol. These two enzymes have different cellular localizations. ADH is located in the cytosol of hepatocytes, whereas CYP2E1 belongs to the microsomal ethanol oxidizing system. They oxidize alcohol to generate acetaldehyde, which is a toxic product of alcohol metabolism and has been implicated in alcohol-induced oxidative stress and hepatotoxicity ^[8]^. Acetaldehyde can be further oxidized to acetate by the mitochondrial enzyme aldehyde dehydrogenase 2 (ALDH2).

Epidemiological studies have shown that genetic polymorphism of alcohol-metabolizing enzymes, including ADH ^[9–11]^, CYP2E1 ^[11–13]^, and ALDH2 ^[14–16]^, is associated with increased susceptibility to alcohol-induced advanced liver injury. In addition, accumulating evidence suggests that manipulating the expression of alcohol-metabolizing enzymes in experimental animals affects the severity of liver damage induced by alcohol overconsumption. For example, alcohol-fed transgenic mice overexpressing human CYP2E1 have higher serum alanine aminotransferase (ALT) levels and more liver injury ^[17,18]^. In contrast, CYP2E1 inhibitors show protective effects against alcohol-induced liver damage in rats ^[19–22]^. Moreover, ALDH2 deficiency is associated with an increased risk of liver cancer in patients with alcoholic cirrhosis and in mice treated with alcohol and carbon tetrachloride ^[16]^.

Previous studies have suggested that some nutrients affect the development of ALD by regulating the expression of alcohol-metabolizing enzymes ^[23,24]^. To investigate whether dietary cholesterol modulates alcohol metabolism, López-Islas et al ^[25]^ isolated primary hepatocytes from high-cholesterol diet-fed mice and found enhanced CYP2E1 content and activity. Interestingly, when hepatocytes are exposed to combined dietary cholesterol and alcohol treatment, there is no further increase in CYP2E1 expression despite that alcohol alone also upregulates hepatic CYP2E1 expression ^[25,26]^. Consistent with this in vitro study, liver samples from mice chronically fed a liquid diet containing alcohol (27% of calories) and 0.5% (w/v) cholesterol show a similar expression of CYP2E1 compared with that of mice on an alcohol-only diet ^[27]^. Moreover, combined ethanol and cholesterol consumption does not alter the hepatic expression of ADH and ALDH2 ^[27]^.

## 3. Combined feeding of alcohol and dietary cholesterol on liver damage

### 3.1 The effect of heavy drinking and high-cholesterol diet on fatty liver development

To investigate the combined effect of dietary cholesterol and alcohol on the development of hepatic steatosis, Krishnasamy et al ^[27]^ added cholesterol (0.5%; w/v) to the widely used Lieber-DeCarli liquid diets. Male C57BL/6J mice were fed one of the four types of liquid diets (control, EtOH, control + cholesterol, EtOH + cholesterol) chronically for 3 months. They found that the addition of cholesterol to an alcohol-containing liquid diet causes significant increases in liver triglyceride contents and circulating ALT levels, which were much higher than those of mice fed the diet containing alcohol or cholesterol alone. In addition, pathological analysis of mouse livers from combined feeding reveals lipid droplets accumulation in hepatocytes ^[27]^. Similar histological findings were reported by Li et al ^[28]^ in the Institute of Cancer Research (ICR) male mice fed the combined diet for 5 weeks. The accumulation of hepatic triglyceride could be resulted from the imbalance between lipid influx/synthesis and lipid export/degradation. Fatty acid synthase (FASN) and carnitine palmitoyltransferase 1 (CPT1) are two critical enzymes affecting hepatic triglyceride contents, which control the de novo fatty acid synthesis and mitochondrial fatty acid oxidation, respectively. Reduced expression of FASN and CPT1 are observed in mice fed EtOH + cholesterol diet, indicating that decreased degradation of fatty acids, but not endogenous lipogenesis, could be a major contributor to the development of hepatic steatosis induced by combined feeding ^[27]^. Enhanced fatty acid uptake and reduced very low-density lipoprotein (VLDL) particle secretion have been shown to participate in the development of alcoholic steatosis. Specifically, excessive alcohol intake upregulates hepatic expression of cluster of differentiation 36 (CD36), a membrane protein responsible for fatty acid uptake ^[29]^. Moreover, CD36 deficiency attenuates ethanol-induced hepatic lipid accumulation in mice ^[30]^. Reduced VLDL particle assembly and production have been reported in chronic alcohol-fed rats ^[31,32]^. However, whether altered hepatic fatty acid uptake and/or VLDL production is involved in dietary cholesterol-induced exacerbation of alcoholic fatty liver disease has not been investigated yet.

### 3.2 The effect of heavy drinking and high-cholesterol diet on hepatic inflammation and cytotoxicity

It has been reported that approximately 10% to 35% of the patients who continuously drink excessive alcohol develop alcoholic steatohepatitis, which is characterized by increased inflammation and enhanced immune cell infiltration in the liver ^[2]^. Consistently, experimental rodents chronically fed alcohol-containing liquid diets exhibit increased hepatic inflammatory responses ^[33,34]^. Notably, in the presence of dietary cholesterol, chronic alcohol feeding causes an even greater increase in hepatic leukocyte infiltration, which is associated with much higher expression of inflammatory markers in the liver, such as high-mobility group box 1, toll-like receptor 4, tumor necrosis factor-alpha (TNFα) and nuclear factor кB ^[27,28]^. This exacerbated hepatic inflammation could be resulted from the hepatic accumulation of cholesterol ^[27,28]^, which activates Kupffer cells to trigger an inflammatory response and lead to the production of inflammatory cytokines and chemokines, attracting the infiltration of monocytes, neutrophils, and other immune cells ^[35]^. Indeed, elevated expression of F4/80 and myeloperoxidase, indicators of macrophages and neutrophils respectively, is reported in mouse livers exposed to the combined diet containing excessive alcohol and cholesterol ^[27]^.

Increasing lines of evidence suggest that oxidative and endoplasmic reticulum (ER) stresses are significantly induced in alcohol-fed rodents and heavy drinkers ^[36–38]^. Of note, compared with mice fed alcohol only or cholesterol only diet, mice on EtOH + cholesterol diet show the greatest increases in the hepatic expression of 4-hydroxynonenal adducts, an indicator of oxidative stress ^[27]^. Moreover, elevated ER stress is observed in mouse livers and primary hepatocytes exposed to both cholesterol and alcohol ^[25]^. The hepatotoxicity could be due to cholesterol-mediated alterations in membrane dysfunction. As one of the structural components of the cell membrane, cholesterol plays an important role in determining membrane rigidity and affecting the function of membrane proteins. An increase in membrane cholesterol content has been shown to promote the generation of mitochondrial reactive oxidative species and activate unfolded protein response/ER stress ^[35]^. In particular, mitochondrial free cholesterol can sensitize hepatocytes to inflammatory response and cause cytotoxicity ^[39]^. However, the changes of free cholesterol content following combined feeding have not been reported yet. Although the alcohol-induced increase in hepatic cholesterol occurs mainly in the esterified form ^[40]^, it is possible that dietary cholesterol would promote a slight accumulation of free cholesterol in alcohol-fed animal models and this small elevation of hepatic free cholesterol could significantly affect the mitochondrial function and lead to hepatotoxicity.

### 3.3 The effect of heavy drinking and high-cholesterol diet on fibrotic liver damage

Despite the observed alcoholic cirrhosis in heavy drinkers, this pathological change is difficult to be recapitulated in animal models fed an alcohol-only diet ^[41]^. By feeding an alcohol-containing liquid diet for 3 months, Krishnasamy et al ^[27]^ observed that male C57BL/6J mice exhibit increased hepatic expression of type I collagen, transforming growth factor β1 (TGF β1) and smooth muscle α-actin. Notably, when 0.5% cholesterol is added to the alcohol diet, these fibrotic markers are greatly enhanced in mouse livers, together with dramatically increased blue Mason’s Trichrome staining in liver sections ^[27]^. These findings indicate that combined feeding stimulates hepatic stellate cells probably via the enhanced expression of fibrogenic cytokines, such as TNFα and TGFβ, produced by activated Kupffer cells following hepatic cholesterol accumulation. Moreover, a recent publication has demonstrated that free cholesterol in hepatic stellate cells can directly activate these cells and contribute to liver fibrosis development ^[42]^. However, the effect of dietary cholesterol on hepatic free cholesterol content and its accumulation in hepatic stellate cells in alcohol-fed experimental animals remains to be determined.

## 4. Combined alcohol and dietary cholesterol feeding on cholesterol metabolism

### 4.1 The effect of alcohol only and combined feeding on hepatic cholesterol metabolism

Cholesterol homeostasis is well controlled in the body via the balance between intestinal cholesterol absorption, endogenous cholesterol synthesis, and hepatic cholesterol excretion. Cholesterol absorption from the small intestine is mediated by Niemann-Pick C1-Like 1 (NPC1L1), a transmembrane protein localized to the apical side of enterocytes. Absorbed cholesterol in the small intestine is incorporated into the chylomicron and delivered to the liver. Cholesterol can be synthesized in almost all cell types. The liver accounts for about 50% of cholesterol biosynthesis, in which 3-hydroxy-3-methylglutaryl-coenzyme A reductase (HMGCR) is one of the rate-limiting enzymes in endogenous cholesterol production. Uptake of cholesterol-rich low-density lipoprotein (LDL) particles through LDL receptor (LDLR)-facilitated endocytosis significantly contributes to the cholesterol pool in the liver. Hepatic cholesterol can be assembled into VLDL and secreted to circulation. Cholesterol cannot be degraded in the body. Excess cholesterol in peripheral tissues is delivered to the liver via high-density lipoprotein-mediated reverse cholesterol transport for excretion. Conversion of cholesterol to bile acids also helps the removal of excess cholesterol.

In 1970s, scientists used liver and intestinal slices obtained from alcohol-fed rats and showed that alcohol feeding promotes endogenous cholesterol synthesis ^[43,44]^. The role of alcohol in regulating cholesterol biosynthesis is also reported in ICR mice and Sprague Dawley (SD) rats after chronic alcohol feeding, showing increased hepatic expression of sterol regulatory element-binding transcription factor 2 (SREBP2) and HMGCR ^[28,45]^. Interestingly, when dietary cholesterol is combined with alcohol feeding, a greater accumulation of hepatic cholesterol is observed than that induced by cholesterol alone or alcohol alone ^[43]^. In support of this finding, two independent studies have shown that combined alcohol and cholesterol feeding lead to the most cholesterol accumulation in mouse livers ^[27,28]^. Dietary cholesterol feeding usually suppresses endogenous cholesterol synthesis and upregulates bile acid production as feedback mechanisms to reduce the hepatic burden of cholesterol accumulation. However, the hepatic expression of SREBP2, HMGCR, CYP7A1, and adenosine triphosphate (ATP)-binding cassette (ABC) subfamily G member 5 (ABCG5) is similar between mice fed a combined diet and an alcohol-only diet. This suggests that the effect of alcohol on the elevation of cholesterol biosynthesis and the suppression of bile acid metabolism is dominant ^[43]^, leading to further enhanced hepatic cholesterol accumulation in mice fed a combined diet. In addition, increased intestinal expression of NPC1L1 is observed in mice on a combined diet compared with that of mice on the alcohol-only diet. Detailed cellular mechanisms by which alcohol affects cholesterol absorption and synthesis in the presence of exogenous cholesterol need to be thoroughly investigated.

### 4.2 Effect of alcohol only and combined feeding on plasma cholesterol

The development of hypercholesterolemia in alcohol-drinking subjects and experimental rodent models has been reported ^[45,46]^. This could be due to increased cholesterol intake and cholesterol biosynthesis as well as decreased cholesterol excretion in the form of bile salts ^[28,43,45]^. When ethanol and cholesterol feeding are combined, significantly increased plasma total cholesterol and LDL cholesterol (LDL-C) levels are observed in mice compared with that of mice on alcohol-only diet ^[28]^.

## 5. The potential role of cholesterol-lowering drugs in the treatment of ALD

Statin is the first class of plasma LDL-C-reducing pharmacotherapy by inhibiting HMGCR and promoting hepatic cholesterol uptake. Although both clinical trials and animal studies have reported a slight elevation of serum ALT levels following long-term statin treatment, chronic use of atorvastatin did not increase serum ALT concentrations in alcohol-treated rats ^[47]^. When experimental animals are fed a combined diet containing alcohol and dietary cholesterol, the greatest accumulation of hepatic cholesterol and dramatic elevation of plasma ALT are observed ^[27,28]^. However, it is largely unknown whether statin treatment would prevent and/or attenuate liver damage induced by joint feeding. In addition, the potential increase in ALT and other indicators related to liver function should be monitored closely.

Ezetimibe, commercially known as Zetia, has been used to reduce LDL-C in hypercholesterolemia patients ^[48]^. Ezetimibe dramatically lowers plasma cholesterol levels and prevents the development of atherosclerosis in rodents by inhibiting the function of intestinal NPC1L1 and limiting cholesterol absorption from the small intestine. In addition to the role of ezetimibe in regulating cholesterol metabolism, accumulating evidence has demonstrated that ezetimibe treatment alleviates fatty liver disease and hepatitis in obese animals ^[49–52]^. Alcohol has been shown to increase intestinal cholesterol absorption in rodent models ^[44]^. In addition, increased intestinal expression of NPC1L1 is reported in mice fed combined alcohol and cholesterol diet ^[28]^. These observations lead to the investigation of whether ezetimibe could reverse the synergistic adverse effects of alcohol and cholesterol on liver damage. Recently, Li et al ^[28]^ reported that supplementation of ezetimibe dramatically suppresses the elevation of plasma cholesterol levels and attenuates the development of liver injuries induced by combined high cholesterol and alcohol feeding in rats.

Clinical trials have demonstrated the successful use of anti-proprotein convertase subtilisin/kexin type 9 (PCSK9) antibodies in lowering blood cholesterol levels and the treatment of cardiovascular disease ^[53,54]^. PCSK9 is mainly expressed in the liver and plays a critical role in regulating LDLR expression and affecting plasma LDL-C levels. An epigenome-wide association study of individuals with alcohol use disorder reveals that the expression of PCSK9 is regulated by alcohol consumption ^[55]^. In addition, Lee et al ^[56]^ reported that chronic anti-PCSK9 treatment by Alirocumab (Praluent®), one of the PCSK9 monoclonal antibodies, attenuates alcohol-induced hepatic steatosis and liver injury in rats. Interestingly, this PCSK9 antibody does not alleviate alcohol-induced increases in hepatic and plasma cholesterol levels ^[56]^. Nevertheless, it is interesting to investigate whether PCSK9 inhibition could protect alcohol-exposed animals from liver damage in the presence of dietary cholesterol.

## 6. Perspectives

We have summarized the important role of dietary cholesterol in exacerbating ALD (Table 1) and the protective effect of cholesterol-lowering drugs on alleviating liver damage induced by combined feeding of alcohol and cholesterol (Table 2) in rodents. Clinical evidence is required to provide valuable epidemiological data and therapeutic strategies. The pathological role of dietary cholesterol in the development and progression of ALD and nonalcoholic fatty liver disease (NAFLD) is quite similar ^[5,35]^. Considering the promising role of cholesterol-reducing medications in the treatment of NAFLD ^[5]^, it is highly possible that these drugs could be used to combat ALD in subjects consuming high amount of dietary cholesterol.

**Table 1.** Summary of rodent models fed the combined diet containing both alcohol and cholesterol.

Animal model and gender	Alcohol-containing diet	Cholesterol content in the diet	Duration of diet feeding	Liver damage examined	Cholesterol metabolism examined	References
C57BL/6J mice; male	Lieber-DeCarli liquid diets containing 27% of calories from ethanol	0.5% cholesterol (w/v)	3 months	Serum alanine aminotransferase (ALT), hepatic steatosis, liver inflammation, and fibrosis	Hepatic total cholesterol	Krishnasamy et al 2016 ^[27]^
Institute of Cancer Research (ICR) mice; male	Lieber-DeCarli liquid diets containing 28.8% of calories from ethanol (equivalent to 4% alcohol; v/v)	0.5% cholesterol (w/v)	5 weeks	Serum ALT and aspartate aminotransferase (AST), hepatic steatosis, and liver inflammation	Serum total cholesterol, low-density lipoprotein cholesterol, high-density lipoprotein cholesterol, and hepatic total cholesterol	Li et al 2019 ^[28]^
Sprague Dawley rats; male	22% alcohol (v/v) in drinking water	Not mentioned	16 weeks	Serum ALT and AST, hepatic lipid droplet accumulation, and liver fibrosis	Serum total cholesterol	Li et al 2019 ^[28]^

**Table 2.** Summary of cholesterol-lowering drugs in the treatment of ALD in rodent models.

Animal model and gender	Alcohol-containing diet	Cholesterol-lowering drugs	Duration of diet feeding	Liver damage examined	Cholesterol metabolism examined	References
Wistar elevated plus-maze (EPM)-1 rats; male	10% alcohol (v/v) in drinking water	Atorvastatin (0.8 mg/kg; 5 days per week by oral gavage)	2 months	Alanine aminotransferase (ALT), aspartate aminotransferase (AST), and lactic dehydrogenase levels in liver perfusates	Not examined	Ito et al 2007 ^[47]^
Sprague Dawley rats; male	22% alcohol (v/v) in drinking water	Ezetimibe (1 mg/kg; daily by oral gavage)	16 weeks of diet feeding with the addition of ezetimibe for the last 12 weeks	Serum ALT and AST, hepatic lipid droplet accumulation, and liver fibrosis	Serum total cholesterol	Li et al 2019 ^[28]^
Sprague Dawley rats; male	A liquid diet containing 12% (v/v) alcohol	PCSK9 inhibitor alirocumab (50 mg/kg; weekly by subcutaneous injection)	6 weeks with 5-day liquid diet exposure and 2-day regular chow over the weekends	Serum ALT and AST, hepatic triglyceride content, liver inflammation, and fibrosis	Serum and hepatic total cholesterol	Lee et al 2019 ^[56]^

PCSK9, proprotein convertase subtilisin/kexin type 9.

Elevated blood alcohol and cholesterol levels are recognized as independent risk factors in the development of cerebrovascular disease ^[58,59]^. Surprisingly, Bukiya et al ^[58]^ reported that reduced artery cholesterol content exacerbates alcohol-induced constriction of cerebral arteries ^[60]^. These findings demonstrate the protective role of dietary cholesterol in preventing alcohol-induced cerebral artery constriction ^[60]^. Consistently, clinical trials suggest that excessive alcohol consumption is not a risk factor for cerebral ischemia in Japanese hypercholesterolemic patients ^[61,62]^. Therefore, the potential development of cerebral artery constriction in alcohol-consuming subjects treated with cholesterol-lowering medications needs to be considered and carefully monitored.

Liver injury is highly associated with the development of insulin resistance. Indeed, the link between excessive drinking and decreased insulin sensitivity has been established in heavy drinkers and alcohol-exposed rodents ^[57,63,64]^. Interestingly, excessive intake of cholesterol has been shown to be an independent risk factor to induce glucose intolerance in healthy subjects ^[65]^. In addition, rodents develop insulin resistance in a dietary cholesterol concentration-dependent manner ^[66]^. By blocking intestinal cholesterol absorption, ezetimibe improves glucose tolerance in obese animal models ^[49,67,68]^. However, the role of cholesterol in alcohol-associated dysregulation of insulin signaling has not been investigated yet.

Previous findings have demonstrated the pro-inflammatory effects of cholesterol in tissue damage and disease development ^[69,70]^. In particular, Progatzky et al ^[71]^ reported that mice exposed to a high-cholesterol diet develop inflammation in the intestines. This local inflammation could lead to enhanced gut permeability and facilitate the translocation of intestinal bacterial products. Since the composition of gut microbiota can be strongly affected by excessive alcohol intake ^[72]^ and alcohol-induced dysbiosis is closely related to liver damage, it is interesting to explore the adverse effect of dietary cholesterol on the gut-liver axis in the exacerbation of ALD.

## Conflicts of interest

The author declare that she has no conflicts of interest.

## Funding

This research was supported by grants to L.J. from the National Institute of Alcohol Abuse and Alcoholism (NIAAA) of the National Institutes of Health (NIH) (K01AA024809 and R03 AA028585). This project was partially funded by The University of Texas at Dallas Office of Research and Innovation through the New Faculty Research Symposium Grant Program.
